# STAT6 Is Critical for the Induction of Regulatory T Cells In Vivo Controlling the Initial Steps of Colitis-Associated Cancer

**DOI:** 10.3390/ijms22084049

**Published:** 2021-04-14

**Authors:** Yael Delgado-Ramirez, Angel Ocaña-Soriano, Yadira Ledesma-Soto, Jonadab E. Olguín, Joselín Hernandez-Ruiz, Luis I. Terrazas, Sonia Leon-Cabrera

**Affiliations:** 1 Unidad de Biomedicina, Facultad de Estudios Superiores-Iztacala, Universidad Nacional Autónoma de México, Tlalnepantla, Edo. De México 54090, Mexico; sonr_sanjlv@hotmail.com (Y.D.-R.); angelaos_7@outlook.com (A.O.-S.); lesy_790413@yahoo.com.mx (Y.L.-S.); je.olguin@iztacala.unam.mx (J.E.O.); literrazas@unam.mx (L.I.T.); 2 Laboratorio Nacional en Salud, Facultad de Estudios Superiores-Iztacala, Universidad Nacional Autónoma de México, Tlalnepantla, Edo. De México 54090, Mexico; 3 Servicio de Farmacología Clínica, Hospital General de México Dr. Eduardo Liceaga, Mexico City 06720, Mexico; hernandezjoselin@hotmail.com; 4 Carrera de Médico Cirujano, Facultad de Estudios Superiores Iztacala, Universidad Nacional Autónoma de Mexico, Tlalnepantla, Edo. De México 54090, Mexico

**Keywords:** STAT6, colorectal cancer, regulatory T cells, colitis-associated-cancer

## Abstract

Inflammation is the main driver of the tumor initiation and progression in colitis-associated colorectal cancer (CAC). Recent findings have indicated that the signal transducer and activator of transcription 6 (STAT6) plays a fundamental role in the early stages of CAC, and STAT6 knockout (STAT6^−/−^) mice are highly resistant to CAC development. Regulatory T (Treg) cells play a major role in coordinating immunomodulation in cancer; however, the role of STAT6 in the induction and function of Treg cells is poorly understood. To clarify the contribution of STAT6 to CAC, STAT6^−/−^ and wild type (WT) mice were subjected to an AOM/DSS regimen, and the frequency of peripheral and local Treg cells was determined during the progression of CAC. When STAT6 was lacking, a remarkable reduction in tumor growth was observed, which was associated with decreased inflammation and an increased number of CD4+CD25+Foxp3+ cells in the colon, circulation, and spleen, including an over-expression of TGF-beta, IL-10, and Foxp3, compared to WT mice, during the early stages of CAC development. Conversely, WT mice showed an inverse frequency of Treg cells compared with STAT6^−/−^ mice, which was followed by intestinal tumor formation. Increased mucosal inflammation, histological damage, and tumorigenesis were restored to levels observed in WT mice when an early inhibition/depletion of Treg cells was performed in STAT6^−/−^ mice. Thus, with STAT6 deficiency, an increased number of Treg cells induce resistance against tumorigenesis, arresting tumor-promoting inflammation. We reported a direct role of STAT6 in the induction and function of Treg cells during CAC development and suggest that STAT6 is a potential target for the modulation of immune response in colitis and CAC.

## 1. Introduction

Colorectal cancer (CRC) is the third most commonly diagnosed neoplasm, with the second highest cancer-associated mortality rate in the world [1]. In 2018, a total of 881,000 deaths associated with CRC were reported, and 1.8 million newly diagnosed cases were registered worldwide [2]. Epidemiological studies suggest that chronic inflammatory processes, such as ulcerative colitis (UC) and Crohn’s disease (CD), are associated with the risk of developing colitis-associated colorectal cancer (CAC). The relationship between inflammation-dysplasia and cancer in CAC has been well established, supporting the idea that chronic inflammation is the main driving force associated with carcinogenesis [3,4].

The signal transducer and activator of transcription 6 (STAT6) is a member of the STAT family of proteins formed by seven transcription factors involved in cytokine-related signaling [5] In particular, STAT6 participates in the cellular response to interleukin- (IL-) 4 and IL-13 and it is involved in the generation of CD4+ Th2 cells [5]. Recently, STAT6 signaling has been associated with the initiation of malignant transformation and tumor establishment, and STAT6-phosphorylation has frequently been found in malignant cells that regulate several genes crucial for the immune response and proliferation [6]. The persistent activation of STAT6 has been observed in the development of prostate, breast, and colon carcinomas [7,8,9]. Patients with colorectal cancer exhibit a significant STAT6 activity in the colonic epithelium, and STAT6 expression is associated with lower survival rates, lymph node metastasis, changes in the epithelial barrier function, and alterations in the inflammatory response [9,10,11,12].

Previously, we demonstrated that STAT6 plays important roles in the early stages of CAC, and it modulates inflammatory responses, while controlling cell recruitment and proliferation in the colon [13]. The induction of CAC in STAT6-deficient mice (STAT6^−/−^) in an azoxymethane (AOM)/DSS model resulted in a reduced tumorigenicity, associated with reduced inflammation, decreased concentrations of cyclooxygenase-2 (Cox-2) and nuclear β-catenin protein in the colon, and decreased mRNA expression levels of IL-17A and tumor necrosis factor-α (TNF-α) [13]. In addition, the number of circulating inflammatory monocytes and granulocytes was decreased in STAT6^−/−^ mice, suggesting that immunoregulatory mechanisms are involved.

Regulatory T (Treg) cells play a major role in coordinating immunomodulation during CRC [14]. Foxp3 is a transcription factor essential for the initiation and maintenance of the Treg-suppressive phenotype. The infiltration of Treg cells in the colon has been positively correlated with lymph node metastasis and increased degree of malignancy [15]. However, recent studies have found that high densities of intra-tumoral Foxp3(+) T cells in an earlier CRC stage showed a positive correlation with overall survival [16,17,18,19], and distinct subpopulations of tumor-infiltrating Foxp3(+) T cells contribute in opposing ways to the determination of CRC prognosis [20]. In an experimental CAC model, the frequency and suppressive capacity of Treg cells and the expression of Tim-3, PD-1, and CD127 molecules were increased in late-stage of CAC [21]. Conversely, in early CAC, a reduced percentage of Treg cells and decreased expression of molecules that correlate with suppression were detected in the blood and spleen [21], suggesting that Treg cells could modify their phenotype depending on the grade of alterations in CRC.

STAT6 is implicated in the regulation of T cell proliferation and in the activity of Treg cells through a direct interaction with the transcription factor, Foxp3. Mice expressing an active form of STAT6 have reduced T cell numbers [22]. Indeed, in a model of TCR transgenic mice, the absence of STAT6 impaired the generation of antigen-specific CD4+CD25+Foxp3+ cells, indicating the role of the STAT6 pathway in the induction of Foxp3 expression [23]. A highly conserved STAT-binding site, located in the first intron of the FOXP3 gene, has been reported [24], and a chromatin immunoprecipitation assay identified a silencing region in the Foxp3 transcript, with a specific binding site for STAT6, preventing Foxp3 mRNA expression [25]. In a murine model of allergic lung inflammation, STAT6^−/−^ mice were highly resistant to airway eosinophilia, which is correlated with a high number of Treg cells in the lungs and spleens, compared to wild-type animals [26]. The depletion of Treg cells partially restores airway inflammation and remodeling in STAT6^−/−^ mice [26]. Together, these findings suggest that STAT6 may play a critical role regulating the function of Treg cells. Thus, the effects of STAT6 on Treg cells in CAC need to be further elucidated.

We have shown that STAT6 deficiency prevents tumorigenesis in an AOM/DSS mouse model by reducing the infiltration of inflammatory cells and down-regulating inflammatory mediators in a disease stage-dependent manner, and we have raised the possibility that STAT6 could also play a yet undetermined function in the development of Treg cells in CAC progression. In the present study, we evaluated the frequency of peripheral and local Treg cells in the course of CAC during STAT6 deficiency and analyzed the effect of Treg cells depletion in the early stages of tumor progression. Interestingly, we found that tumor growth was restored in STAT6^−/−^ mice with the Treg cells reduction, and extensive chronic inflammation was reestablished. Thus, the STAT6 pathway is critical for modulating the activity of Treg cells in the early stages of CAC. With STAT6 deficiency, increased Treg populations induce resistance against tumor-promoting inflammation, and tumorigenesis is therefore stopped.

## 2. Results

### 2.1. The Absence of STAT6 Increases the Number of CD4+CD25+Foxp3+ Cells in Circulation and Spleen during the Early Stages of CAC Development

**Figure 1:**
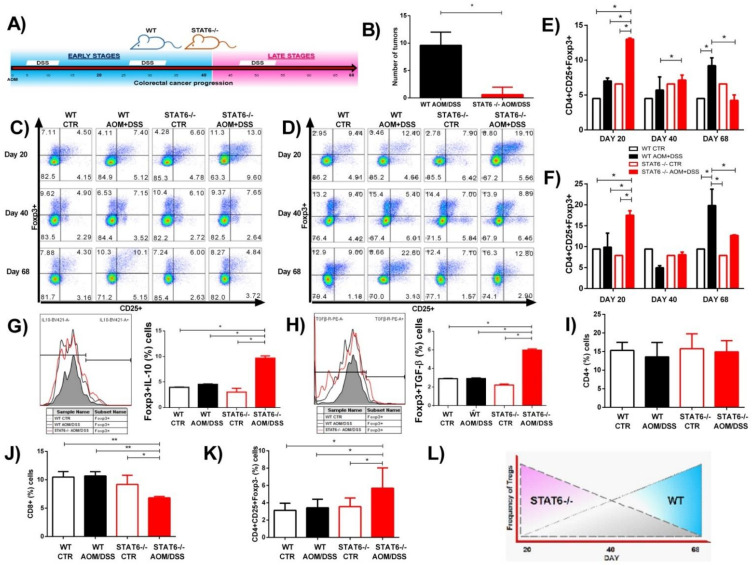
STAT6^−/−^ animals display twice the percentage of CD4+CD25+Foxp3+ Treg cells during early colitis-associated colorectal cancer (CAC) development. (**A**) Schematic time schedule of azoxymethane (AOM) and dextran sodium sulfate (DSS) administration in WT and STAT6^−/−^ mice. After the initial AOM injection (12.5 mg/kg), DSS was given in drinking water for 7 days, followed by 14 days of regular drinking water. The mice were sacrificed on Day 20, Day 40 (early tumor development), and Day 68 (late tumor development), after an AOM injection. (**B**) The number of colorectal tumors in the WT and STAT6^−/−^ mice 68 days after the AOM/DSS administration. The circulating and spleen cells were obtained from the WT or STAT6^−/−^ AOM/DSS-treated or control mice at the indicated time intervals and were analyzed for the expression of Foxp3 and CD25 in living CD4+ cell populations by flow cytometry. Representative dot plots in total blood (**C**) and in splenocytes (**D**). (**E**,**F**) The frequencies of CD4+CD25+Foxp3+ cells in the blood (**E**) and splenocytes (**F**). (**G**,**H**) IL-10 (**G**) and TGF-β (**H**) expressions in CD4+CD25+Foxp3+ cells in the splenocytes of the WT or STAT6^−/−^ AOM/DSS-treated or control mice. Representative histogram plots are shown, as well as graphs showing the percentages of positive cell populations. (**I**–**K**) The frequencies of total CD4+ cells (**I**), CD8+ T cells (**J**), and CD4+CD25+Foxp3- (activated cells) (**K**) in the peripheral blood at Day 20. (**L**) The inverse frequency of Treg cells in the WT or STAT6^−/−^ AOM/DSS-treated animals as CAC progressed. The data are expressed as the mean ± SEM and are representative of two independent experiments with at least three mice per group per day of the analysis. * *p* < 0.05, ** *p* < 0.01.

Previously, we determined the role of STAT6 in the development of CAC using the azoxymethane (AOM)/dextran sodium sulfate (DSS) model [13]. STAT6-deficient mice display high resistance to CAC development. The reduced tumorigenicity was associated with diminished inflammation, without changes in the number of goblet cells, and a decreased mRNA expression of IL-17A and TNFα but increased IL-10 expression in early CAC (Day-20), compared to WT mice [13]. Considering that the inflammation is the main driver of tumor initiation in CAC, one potential mechanism contributing to the suppression of the inflammatory response in STAT6^−/−^ mice may be an increased recruitment of Treg cells. Patients with more Foxp3+ cells in CAC tended to have a better prognosis [18]. To test this hypothesis, we subjected WT and STAT6^−/−^ mice to an AOM/DSS regimen and analyzed the CAC progression at Day 20, Day 40 (early stages), and Day 68 (late stage of tumor development, where adenoma-like lesions are observed) as an approximation of different stages of tumor progression (Figure 1A). As expected, the WT mice displayed both increased numbers of tumors as well as increased tumor load at Day 68 (9.6 ± 2.4), whereas only 30% of STAT6^–/–^ animals developed tumors, and they were scarce (0.6 ± 1.3, *p* < 0.05) (Figure 1B). Next, we analyzed the kinetics of Treg cells (CD4+CD25+Foxp3+) at Day 20, Day 40, and Day 68 after AOM injection by flow cytometry in blood and spleens (Figure 1C,D). In the WT AOM/DSS animals, Treg cells were maintained at a similar frequency as those in control animals in the blood and spleen at Day 20 and Day 40. However, remarkably, a significant increase in the frequency of Treg cells was observed at Day 68, compared to the control and STAT6^−/−^ AOM/DSS mice (9.44 ± 0.1 vs. 19.85 ± 3.8, *p* < 0.05; 12.7 ± 0.1 vs. 19.85 ± 3.8, *p* < 0.05) (Figure 1C–F). In contrast, we found an increased Treg frequency in the blood and spleen of STAT6^−/−^ AOM/DSS animals at Day 20 (early stages of tumor development), compared to the control and WT AOM/DSS mice (17.5 ± 1 vs. 9.44 ± 0.1, *p* < 0.05; 17.5 ± 1 vs. 9.8 ± 3.3, *p* < 0.05) (Figure 1C–F). There was no difference in the frequency of Treg cells between the STAT6^−/−^ AOM/DSS, WT AOM/DSS, and control mice at Day 40. However, at Day 68, the Treg cells frequency dropped in STAT6^−/−^ AOM/DSS animals to similar levels as those in control mice, which is consistent with the fact that there was a lower tumor load (Figure 1C–F). Given that the secretion of suppressive cytokines, such as TGF-β and IL-10, has been considered a mechanism used by Treg cells to suppress the immune responses [14], we decided to evaluate whether Treg cells isolated from spleens with early-stage CAC development (Day 20) may show a different expression of these cytokines under STAT6 deficiency. As shown in Figure 1G,H, at Day 20 of the CAC progression, we observed a higher expression of TGF-β and IL-10 cytokines in CD4+CD25+Foxp3+ cells (Figure 1H) from STAT6^−/−^ AOM/DSS compared to WT AOM/DSS mice. In addition, the frequency of CD4+ cells at Day 20 in the STAT6^−/−^ AOM/DSS and WT AOM/DSS animals was similar (Figure 1I). However, at this time, the proportion of CD8+ cells was lower in the STAT6^−/−^ AOM/DSS in comparison to the WT AOM/DSS mice (Figure 1J). At Day 20, the number of CD4+CD25+Foxp3- cells was higher in STAT6^−/−^ AOM/DSS mice compared to WT AOM/DSS animals (Figure 1K).

Therefore, we detected an inverse frequency of Treg cells between WT AOM/DSS and STAT6^−/−^ AOM/DSS mice as CAC progressed (Figure 1L). At early stages of the CAC induction, tumor-bearing WT mice only reached 9.8% of Treg cells, while STAT6^−/−^ AOM/DSS mice reached 17.5% at the same time point of analysis. However, as CAC progressed, the percentage of Treg cells dropped significantly in the STAT6^−/−^ AOM/DSS (12.7%) animals but was significantly increased in the WT AOM/DSS mice (19.85%) (Figure 1L). These results suggest that STAT6 may influence the induction of Treg cells in vivo in the initial stages of CAC development.

### 2.2. STAT6 Deficiency Increase the Accumulation of Treg Cells in the Colon in Early CAC

**Figure 2:**
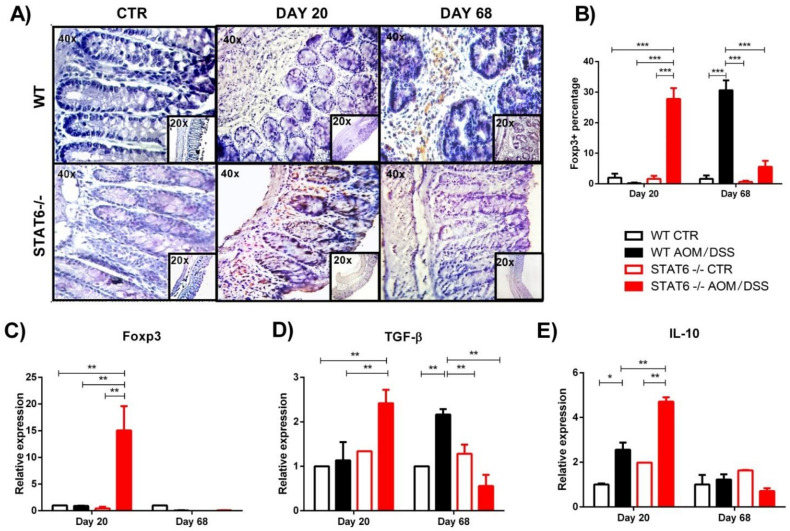
A more pronounced expression of the Foxp3 protein and mRNA in the colon of STAT6^−/−^ AOM/DSS-treated animals was observed during early CAC development. (**A**) A representative example of Foxp3 expression evaluated immunohistochemically in intestinal biopsies from the WT or STAT6^−/−^ AOM/DSS-treated or control mice at Day 20 and Day 68 of the AOM/DSS administration. The sections of tissue were analyzed at 20× and 40× using an optical microscope (**B**) The average percentages of Foxp3+ cells in the colon at Day 20. The quantification of Foxp3+ cells was performed using ImageJ software v.1.48 by counting cells in 10 high-powered fields, with at least three slides per animal. (**C**–**E**) The quantitative real-time PCR analysis of the colonic mRNA expression of Foxp3 (**C**), TGF-β (**D**), and IL-10 (**E**) in the WT or STAT6^−/−^ AOM/DSS-treated or control mice at Day 20 of the AOM administration. The data are expressed as the mean ± SEM and are representative of two independent experiments, with at least three mice per group per day of the analysis. * *p* < 0.05, ** *p* < 0.01, *** *p* < 0.001.

The defective colonic inflammatory response observed in the STAT6^−/−^ AOM/DSS mice at early stages of CAC, which is correlated with few tumor developments, led us to examine the local recruitment of Treg cells. We analyzed the colonic protein expression of Foxp3 by immunohistochemistry during early and late stages of colon tumorigenesis. Our analysis revealed a significant increased accumulation of Foxp3+ cells in the colon of the STAT6^−/−^ AOM/DSS mice, compared to the WT AOM/DSS animals, at Day 20 of the CAC progression (27.8 ± 7.8 vs. 0.2 ± 0.05, *p* < 0.001) (Figure 2A,B). In contrast, during the advanced stages of tumor development (Day 68), the Foxp3+ cells were significantly higher in the tumor-bearing WT mice, compared to the STAT6^−/−^ AOM/DSS animals, (30.6 ± 7.3 vs. 5.6 ± 4.2, *p* < 0.001) (Figure 2A,B). Colonic biopsies of the control mice showed barely detectable Foxp3 staining (Figure 2A,B).

Next, we analyzed the colonic mRNA expression of Foxp3. Similar to the immunohistochemistry results, the relative expression of Foxp3 was significantly higher in the STAT6^−/−^ AOM/DSS mice, compared to the WT AOM/DSS mice, at Day 20 (15 ± 6.4 vs. 0.9 ± 0.07, *p* < 0.05) (Figure 2C). No differences were observed in the Foxp3 expression at Day 40 and Day 68 between the groups (Figure 2C).

To corroborate our findings in the peripheral blood and spleen, we analyzed the gene expression in the colon of TGF-β and IL-10 cytokines that promote the suppressive function of Treg cells. We observed a significant increase levels of TGF-β transcripts in the colons of the STAT6^−/−^ AOM/DSS mice, compared to the WT AOM/DSS mice, at Day 20 (2.4 ± 0.5 vs. 1.13 ± 0.71; *p* < 0.001) (Figure 2D). On the contrary, the expression of TGF-β mRNA was increased in the colons of the tumor-bearing WT mice, compared to the STAT6^−/−^ AOM/DSS animals, at Day 68 of the CAC progression (2.1 ± 0.2 vs. 0.5 ± 0.4; *p* < 0.01) (Figure 2D). At day 20, IL-10 levels were also increased in STAT6^−/−^ AOM/DSS mice compared to the WT AOM/DSS mice (4.7 ± 0.2 vs. 2.5 ± 0.5, *p* < 0.01) (Figure 2E). Interestingly, we observed similar IL-10 mRNA transcripts in the colon of both the WT and STAT6^−/−^ AOM/DSS-treated mice during the advanced stages of tumor development (Figure 2E). Taken together, our results demonstrate that STAT6 deficiency promotes the development of Treg cells responses during early CAC.

### 2.3. Treg Cells Depletion during Early CAC Restores Tumor Development in STAT6-Deficient Mice

**Figure 3:**
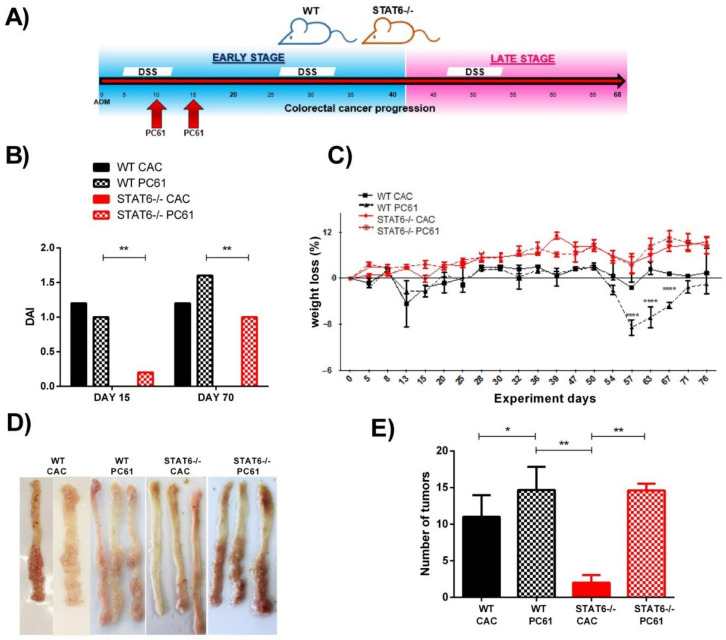
Depletion of Treg cells with anti-CD25 antibody (PC61) during early CAC restores tumor development in STAT6^−/−^ mice. (**A**) A schematic time schedule of the PC61 antibody administration in the WT and STAT6^−/−^ mice that received the AOM/DSS administration (CAC-induced mice). After 10 and 15 days, the groups of WT or STAT6^−/−^ CAC-induced mice were injected i.p. with 200 µg of PC61 antibody. The CAC progression was analyzed over 77 days. (**B**–**E**) The disease activity index (DAI) (**B**), body weight loss (**C**), representative photographs of colons opened longitudinally, showing macroscopic aspects (**D**), and number of tumors (**E**) on Day 77, after the AOM/DSS administration in the WT CAC, WT PC61, STAT6^−/−^ CAC, and STAT6^−/−^ PC61 mice, are shown. The data are expressed as the mean ± SEM and are representative of two independent experiments, with at least five mice per group. * *p* < 0.05, ** *p* < 0.01, **** *p* < 0.001.

We observed that the STAT6^−/−^ mice had twice the Treg cells (CD4+CD25+Foxp3+) in the peripheral blood and spleen than the WT mice during the initial stages of the CAC development. In addition, an increased mRNA and protein Foxp3 expression was observed at Day 20 in the STAT6^−/−^ AOM/DSS colons, which are highly resistant to CAC development. Thus, to investigate the interaction between Treg cells and STAT6 during early CAC, we hypothesized that if the observed increase in Treg numbers was responsible for the resistance of the STAT6^−/−^ mice to tumor development, then the depletion of Treg cells would restore inflammation and tumorigenesis to the levels observed in WT mice. To test this hypothesis, we provided an AOM/DSS regimen to the STAT6^−/−^ and WT mice and at Days 10 and 15, the animals were treated with the PC61 clone of anti-mouse CD25 antibody (Figure 3A). The use of PC61 to deplete Treg cells has been shown previously [27].

Then, we evaluated the CAC progression for 77 days. We monitored the stool consistency and changes in body weight and tumor development. As expected, the WT CAC mice, treated or untreated with PC61 antibody, rapidly showed piloerection and clinical signs of the disease (DAI) throughout the experiment (Figure 3B) and concomitant weight loss (Figure 3C). Conversely, the STAT6^–/–^ CAC mice did not have diarrhea or rectal bleeding during the treatment, compared with the similarly treated WT animals (Figure 3B). However, when the STAT6^–/–^ animals received the PC61 antibody, they showed an increased DAI score, particularly during late CAC development compared to the STAT6^−/−^ CAC mice (Figure 3B). In addition, STAT6^−/−^ PC61 animals showed less body weight loss when compared to the WT CAC and WT PC61 mice (Figure 3C). Interestingly, in the necropsy on Day 77, only 30% of the STAT6^–/–^ CAC mice developed tumors, whereas 100% of the STAT6^−/−^ PC61 animals displayed reddish polypoid tumors in the medial and distal zones of the colon, macroscopic damage, and pathologic alterations, which were similar to the lesions observed in the WT CAC mice (Figure 3D). Additionally, the tumor load in the WT CAC, WT PC61, and STAT6^−/−^ PC61 mice was similar (Figure 3E), demonstrating that STAT6-deficient mice can be made susceptible to CAC development through the early depletion of Treg cells.

### 2.4. Injection of PC61 Antibody during Early CAC in STAT6^−/−^ Mice Promotes Histological Damage

**Figure 4:**
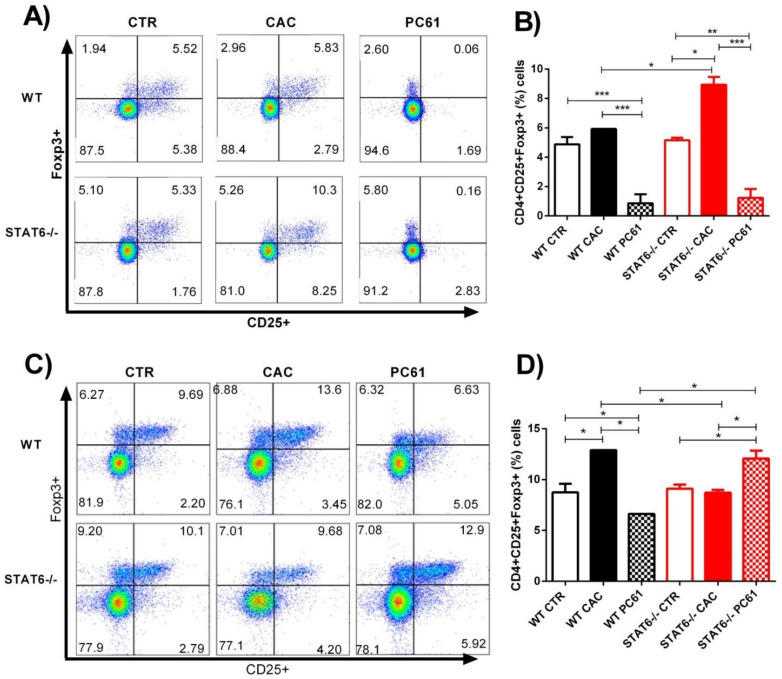
Treatment with anti-CD25 antibody (PC61) during early CAC. The treatment of the mice is shown in Figure 3A. At Day 25 and Day 77, different populations of circulating cells were analyzed by flow cytometry. (**A**) Representative dot plots (**A**,**C**) and frequencies (**B**,**D**) of the CD4+CD25+Foxp3+ cells from the WT CAC or STAT6^−/−^ CAC and PC61-treated WT or STAT6^−/−^ mice at Day 25 (**A**,**B**) or Day 77 (**C**,**D**) after the AOM/DSS administration. The data are expressed as the mean ± SEM and are representative of two independent experiments, with at least three mice per group per day of the analysis. * *p* < 0.05, ** *p* < 0.01, *** *p* < 0.001.

Previous studies have shown that the in vivo depletion of Treg cells by a single injection of the PC61 antibody reaches its peak on Day 8 and remains for 12 days [27]. To validate the use of PC61, we tested the extent of the Treg cell depletion 5 and 71 days after the PC61 antibody administration in the WT and STAT6^−/−^ mice, according to the schedule provided in Figure 3A. In the WT and STAT6^−/−^ CAC-induced mice, around 70% of the CD4+Foxp3+ cells express high levels of CD25, while the remaining cells expressed low levels or no CD25 (Figure 4A, middle panel). In the peripheral blood, five days after the PC61 administration, the CD4+CD25+Foxp3+ cells decreased significantly from 5.9 ± 1.2 to 0.86 ± 0.08, *p* < 0.05 in the WT CAC mice, and from 8.9 ± 1 to 1.2 ± 1, *p* < 0.05 in the STAT6^−/−^ CAC mice (Figure 4A,B). Thus, in response to the PC61 injection, around 75% of the CD4+CD25+Foxp3+ cells were depleted in the peripheral blood, while the CD4+CD25-Foxp3+ cells persisted (Figure 4A, right panel).

Next, we decided to determine if the early depletion of Treg cells during CAC development remained until Day 77, when late-stage tumorigenesis was observed. As shown in Figure 4C,D, the WT CAC mice that did not receive the PC61 antibody showed a significant increased frequency of CD4+CD25+Foxp3+ cells in the peripheral blood, compared to the WT CTR mice (12.9 ± 0.2 vs. 8.7 ± 1.4; *p* < 0.05). However, the WT PC61 mice had a slightly decreased in the frequencies of Treg cells, compared to the WT CTR mice (12.9 ± 0.2 vs. 6.63 ± 0.6; *p* < 0.05). The STAT6^−/−^ CAC mice had similar percentages of Treg cells (8.7 ± 0.6) as the STAT6^−/−^ CTR mice (9.11 ± 0.6) (Figure 4C,D). Interestingly, in the STAT6^−/−^ PC61 mice, there was a significant increase in the frequency of CD4+CD25+Foxp3+ cells, compared to the STAT6^−/−^ CAC mice (12.9 ± 0.2 vs. 8.7 ± 0.6; *p* < 0.05). Additionally, the percentages of Treg cells in the STAT6^−/−^ PC61 mice were similar to those in the WT CAC mice, showing that the use of this antibody during the early stages of carcinogenesis impacted the recruitment of Treg cells under STAT6 deficiency.

**Figure 5:**
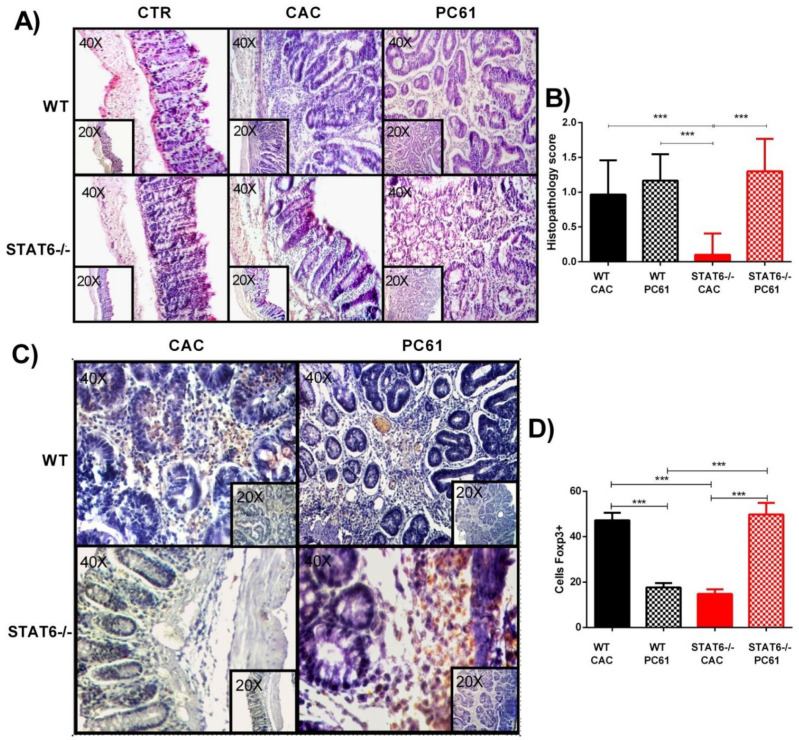
Treatment with anti-CD25 antibody (PC61) favors histological damage in STAT6^−/−^ mice. (**A**) H&E sections and the histological score (**B**) of the colons isolated from the WT CAC or STAT6^−/−^ CAC and PC61-treated WT or STAT6^−/−^ mice at day 77 after the AOM administration. (**C**) A representative example of Foxp3 expression evaluated immunohistochemically in intestinal biopsies of the WT CAC, WT PC61 or STAT6^−/−^ CAC, and STAT6^−/−^ PC61 mice at day 77 after the AOM administration. (**D**) The average percentages of Foxp3+ cells in the colon at day 77. The quantification of Foxp3+ cells was performed using ImageJ software v.1.48 by counting cells in 10 high-powered fields in at least three slides per animal. The sections of tissue were analyzed at 20× and 40× using an optical microscope. The data are expressed as the mean ± SEM and are representative of two independent experiments, with at least three mice per group per day of the analysis. *** *p* < 0.001.

Next, we analyzed the consequences of Treg cells depletion at the local level. Histological analysis revealed that the STAT6^−/−^ PC61 mice displayed intense inflammatory cell infiltration, accompanied by a worse structural integrity, compared to the STAT6^−/−^ CAC mice (Figure 5A). High-grade-dysplasia areas were alternated with normal areas in the intestinal tissue of the STAT6^−/−^ PC61 mice, which was like to the histological damage observed in the WT CAC and WT PC61 mice (Figure 5A,B). Additionally, Foxp3+ cells were clearly observed in the intestine of the WT CAC mice during the late stages of tumor progression (Day 76) (Figure 5C). However, colonic tissue from the WT PC61 mice displayed a significantly lower Foxp3 expression at the same time (Figure 5D). In contrast, the Foxp3+ cells were elevated in the colonic tumors of the STAT6^−/−^ PC61 animals during the late stages of CAC development, compared to the STAT6^−/−^ CAC and WT PC61 mice (Figure 5C,D). Taken together, our results suggest that the Treg cell populations in the tumor microenvironment and peripheral organs prevent tumor progression in STAT6^−/−^ mice.

## 3. Discussion

STAT6 has important roles in the function and activation of immune cells. Here, we showed that STAT6 deficiency is necessary for controlling tumor growth in a model of CAC. Mechanistically, the absence of STAT6 resulted in an increased accumulation of local and peripheral Treg cells and an overexpression of molecules associated with the function of Treg cells during the initial stages of CAC. These data are in accordance with our previous analyses, where the colons of STAT6^−/−^ mice showed a reduction in cell infiltration and decreased production of proinflammatory markers and cytokines in the initial tumor stage [13]. An early depletion of Treg cells during CAC development in STAT6^−/−^ mice restores tumor growth, along with inflammatory infiltration in the colon. These data identify STAT6 as a critical pathway for the induction and function of Treg cells during CAC progression.

IL-4 and IL-13 are canonical inducers of STAT6 activation, when dimers of STAT6 become phosphorylated and are translocated to the nucleus, where they activate or repress target genes. Phospho-STAT6 (p-STAT6) levels have been commonly detected in the colon of patients with clinically detectable CD or UC, and tumoral p-STAT6 is positively correlated to a clinical stage and poor prognosis of human CRC [9,12]. The STAT6 signaling pathway favors the expression of anti-apoptotic proteins [11] and promotes the proliferation of polyp epithelial cells in the colon [12]. Similarly, the persistent activation of STAT6 modifies the expression of proteins involved in epithelial barrier permeability and interrupts tight junction integrity, resulting in the recurrent exposure of luminal microbiota, favoring inflammation and CAC development [12,28]. While these findings suggest the intrinsic importance of STAT6 in regulating tumor growth, our studies have found an important role of STAT6 in non-tumor cells for controlling tumor growth. AOM/DSS administration resulted in a significant decrease in inflammation and tumor development under STAT6 deficiency. In addition, in the STAT6^−/−^ animals, we found a remarkable increase in the frequency of Treg cells during early CAC, relative to the WT mice. The relationship between STAT6 and immune cells was shown to be important in a model of oxazolone-induced colitis, where T cells, macrophages, and natural killer T cells exhibit an increased STAT6 phosphorylation during colitis development [28]. Additionally, ApcMin/+Stat6^−/−^ mice developed few polyps, with a reduced proliferation at the small intestine and MDSCs expansion, decreased PD-1 expression in CD4+ cells, and strong CD8-mediated cytotoxic response [29].

Treg cells have an oncogenic role in tumor progression through the suppression of antitumor immunity and prevention of an active cytotoxic process [30]. Chemokines and cytokines are released in the tumor microenvironment (TME) by cancer cells and tumor stroma-infiltrating cells, leading to the recruitment of Treg cells. In the TME of many tumors, Treg cells suppress effector immune responses, overwhelming the anti-tumor activity mediated by natural killer cells and cytotoxic CD8+ T cells. However, the role of Treg cells in CRC is controversial. Some reports relate Foxp3+ cell infiltration with poor clinical outcomes [31,32,33]. The colonic increase in Foxp3(+) cells is significantly higher in patients with CRC, compared to healthy controls or patients with inflammatory bowel disease [34], and Treg cells with a higher expression of several molecules that correlate with suppression, such as Tim-3, LAG-3, TGF-β, IL-10, CD25, and CTLA-4, are observed in CRC patients [32]. In contrast, a local accumulation and Foxp3 (+) cell density is associated with an improved survival rate and is considered as a good independent prognostic biomarker in the initial stage of colorectal cancers [19,35,36]. The role of Treg cells in CRC seems to be dependent on the co-existence in the tumor tissue and the time of action of different subsets of Foxp3-expressing cells. A study identified two types of Treg cells in CRC, Foxp3^hi^ Treg cells and Foxp3^lo^ non-suppressive T cells [20]. The latter are characterized by secreted inflammatory cytokines along with the instability of Foxp3 and the absence of CD45RA expression, a naive T cell marker [20]. In the present study, we found that in early CAC development, Treg cells were efficiently recruited in STAT6^−/−^ colons, whereas increasing Foxp3 (+) cells in the colon of WT mice were detected only in the late stages of CAC. Interestingly, our previous results for WT mice showed that Treg cells from the late stages of CAC displayed an activated phenotype by expressing PD1, CD127, and Tim-3, along with an increased suppressive capacity in T-CD4+ and T-CD8+ cells. In contrast, Treg cells from WT mice in early CAC were scant and less suppressive [21]. Due to the lack of STAT6^−/−^ Foxp3EGFP reporter mice, we were not able to determine the suppressive capacity of Treg cells in vitro that developed under STAT6 deficiency. However, the immunohistological analyses and H&E staining showed that the STAT6^−/−^ CAC-induced mice displayed decreased inflammatory infiltrate, with less destruction of the intestinal muscle and mucosa, supporting the idea that immunoregulatory mechanisms are taking place. Furthermore, the significant decrease in CD8+ T cells and the positive correlation between the frequency of Treg cells and the transcription levels of Foxp3, TGF-beta, and IL-10 in STAT6^−/−^ colons indicate that the latter are a consequence of the modulating function of Treg cells during CAC progression.

Some authors have suggested that distinct subpopulations of tumor-infiltrating Foxp3 (+) T cells contribute in opposing ways to the determination of CRC prognosis [37,38]. During the early CAC development, epithelial tight junction dysfunction promotes enhanced gut permeability, resulting in the deregulation of the interactions between the intestinal epithelial cells, immune cells, and gut microbiota. Continual exposure to luminal microbiota leads to intestinal inflammation, characterized by the recruitment of innate immune cells, the release of inflammatory mediators, and the subsequent generation and expansion of T-helper 17 (Th17) cells [39]. The local increase of Treg cells in early CAC could suppress or prevent tumor formation through Th17 cell suppression. Here, we use an anti-CD25 monoclonal antibody (PC61) to deplete Treg cells only during early CAC. Interestingly, we observed histological damage and tumor growth recovery during STAT6 deficiency, supporting the idea that Treg cells developed in STAT6^−/−^ mice are protective against colon tumorigenesis. This finding seems to be compatible with that of a recent study showing that the administration of the natural compound, Parthenolide, during experimental colitis significantly relieved colon inflammation and improved the colitis symptoms [40]. The protective effect of this molecule was associated with an increased frequency of colonic Treg cells, a downregulation of the ratio of colonic Th17 cells, together with a more abundant gut microbial diversity and flora composition [40]. One possible reason for the protection observed in the STAT6^−/−^ mice during CAC progression could be the alterations in gut microbiota. Thus, this condition needs further research. Because the balance between Th17/Treg cells and these regulatory factors is decisive in CAC progression, it could be interesting to determine if the tumor growth observed in STAT6^−/−^ mice after Treg depletion is Th17-mediated.

Depending on the grade of alteration during CRC progression, Treg cells may modify their phenotype and exert different effects on cancer progression. Foxp3 (+) cells infiltrating colorectal carcinomas could be associated with their capacity to suppress tumor-promoting inflammatory immune response caused by infectious stimuli from bacterial translocation through the mucosal barrier [41]. In a number of murine models, adoptively transferred Treg cells prevent the onset of colitis or treat established colitis [42,43,44], and the in vitro expansion of Treg cells from the blood of patients with CD is considered as a feasible adoptive cell therapy for this disease [45,46]. The significantly higher percentages of Treg cells found in STAT6^−/−^ CAC-induced colons, compared with those found in WT colons, suggest that STAT6 limits Treg generation and recruitment by undetermined mechanisms. Additionally, a previous study from our laboratory demonstrated that the number of circulating CD11b+Ly6C^hi^CCR2+ monocytes and CD11b+Ly6C^low^Ly6G+ granulocytes was decreased in a STAT6-dependent manner [13]. Additionally, a significant reduction in the expression of the chemokines, CCL9 and CCL25, and the chemokine receptor, CXCR2, both involved in the recruitment of inflammatory cells, was shown in STAT6^−/−^ mice during CAC progression. CCR2 is responsible for the recruitment of Ly6C^hi^ “inflammatory monocytes” to peripheral sites of inflammation, where they display inflammatory, phagocytic, and proteolytic functions [47]. Thus, the STAT6 pathway in Treg cells has an intricate outcome and should be addressed in the context of an inflammatory environment. However, the local increase of Foxp3 (+) cells could be used to suppress antitumor immunity in a final phase of tumor formation. In our lab, when the Treg cells were depleted during the second DSS cycle of experimental CAC in WT mice, a reduction of 50% of Treg cells resulted in a better prognostic value, with a significant reduction in the tumor load [22]. In contrast, in the present study, an earlier Treg depletion slightly increased the tumor load in WT mice. All these results suggest that Treg cells have a dynamic behavior influenced by STAT6 during CAC development.

Recently, a study demonstrated that STAT6 plays a critical role in the generation of Treg cells induced by B cells (Treg-of-B cells) [48]. STAT6 phosphorylation was associated with the capacity of Treg-of-B cells to alleviate inflammation in an animal model of asthma in vivo [48]. In accordance with our results, in an allergic disease model, a high number of Treg cells in the lungs and spleens in STAT6^−/−^ mice, compared to WT animals, were associated with a decreased allergic response [26]. However, it would be interesting to determine if the stability in the expression of Foxp3 and therefore the differentiation and functional properties of Treg cells are modified in a STAT6-dependent manner. Furthermore, the role of STAT6 in modulating different types of Treg cells (natural, induced, type 1 T regulatory cells, and Treg-of-B cells) may be useful to develop new therapeutic strategies for relieving CAC or CRC.

In conclusion, STAT6 seems to play a central role in the regulation of the activity of Treg cells, particularly during the initial stages of CAC development, through the modulation of intense inflammatory responses.

## 4. Materials and Methods

### 4.1. Mice

Eight- to 10-week-old female BALB/c and STAT6^−/−^ mice were purchased from Harlan Laboratories (México City, México) and maintained in a pathogen-free environment at the Facultad de Estudios Superiores Iztacala (FES-I), Universidad Nacional Autónoma de México (UNAM) animal facilities. The animals were fed Purina Diet 5015 and water ad libitum. All experimental procedures were in strict accordance with the recommendations in the Guide for the Care and Use of Laboratory Animals of the National Institutes of Health (USA) and were approved by the Committee on the Ethics of Animal Experiments of the FES-I (UNAM).

### 4.2. CAC Induction

Mice received an intraperitoneal (i.p.) injection of 12.5 mg/kg azoxymethane (AOM) (Sigma, Santa Cruz, CA, USA). Five days later, 2% dextran sulfate sodium (DSS, MW: 40000, (MP Biomedicals, Santa Ana, CA, USA)) in drinking water was administered ad libitum for 7 days. Mice were then provided regular water for 14 days and subjected to two more DSS cycles. To examine the early and late transformative stages in CAC, the mice were slaughtered on day 20 and day 40 (early tumor development), and day 68 or day 77 (late tumor development) after AOM injection. Throughout the experiment, mice were monitored weekly for body weight, stool consistency or diarrhea, and presence of blood in the rectum or stool. The disease activity score (DAI) was calculated as described previously [49]. At the sacrifice, the colon was removed, weighed, and submitted for macroscopic inspection.

### 4.3. Histological Analysis

Longitudinal sections from the large intestine were immediately fixed according to previously described protocols [13]. Colon sections with a thickness of 5 μm were stained with hematoxylin and eosin (H&E) to visualize morphology and inflammatory changes were evaluated in 5 sections from each sample. For immunohistochemical staining, sections were incubated overnight at 4 °C with primary antibodies against Foxp3 (GeneTex, Irvine, CA, USA), and were then developed following a conventional technique. The quantification of Foxp3 (+) cells was performed using ImageJ software v.1.48 by counting cells in 10 high-powered fields, with at least three slides per animal.

### 4.4. Flow Cytometry

Single cell suspensions from spleens and the circulation were obtained during the sacrifice and incubated with anti-CD4, anti-CD8 and anti-CD25 antibodies (BioLeg-end, San Diego, CA, USA) or anti-LAP (TGF beta1) and anti-IL-10 (BD Biosciences, San Jose, CA, USA) for 30 min (4 °C, in the dark). Then, cells were washed and incubated in FOXP3 Fix/Perm Buffer Set (BioLeg-end, San Diego, CA, USA) for 30 min following the manufacturer’s instructions and then stained with anti-Foxp3 antibody (Becton Dickinson, San Jose, CA; USA). Cells were washed, resuspended in PBS, and analyzed on Attune™ NxT Flow Cytometer (ThermoFisher Scientific) cytometer. The flow data were analyzed using flow cytometry analysis software (FlowJo; Tree Star, Inc., Ashland, OR, USA).

### 4.5. RNA Extraction and RT-PCR

Tissues were obtained and processed as previously described [17]. The RNA was purified using TRIzol reagent (Invitrogen, Carlsbad, CA, USA) following the manufacturer’s instructions. One microgram of RNA was used for first-strand cDNA synthesis with RevertAid H Minus First Strand cDNA Synthesis Kit (Thermo Scientific, Rockford, IL, USA). A CFX96 Touch Real-Time PCR Detection System (Bio-Rad, México City, México) and a SYBR Select Master Mix for CFX (Thermo Scientific, Rockford, IL, USA) were used for quantitative real-time RT-PCR analysis. The relative expression levels for a target gene were normalized by GAPDH. Primer sequences used in qRT-PCR analysis are: IL-10F (TGCTGCCTGCTCTTACTGAC), IL-10 R (GGGGCATCACTTCTACCAGG); TGF-β F (GCCCTTCCTGCTCCTCAT), TGF-β R (TTGGCATGGTAGCCCTTG); Foxp3 F (CCTTCTCCAGGACAGA), Foxp3 R (GATCATGGCTGGGTTGT), and GAPDH F (TCACGCCACAGTTTCCCGGAG), GAPDH R (CCTCAAGATCAGCAATGCCT).

### 4.6. Treg Depletion

WT and STAT6^−/−^ mice were injected i.p. with 200 µg/mouse of InVivoMAb anti-mouse CD25 (IL-2Rα) (clone PC-61.5.3) (BioXcell, West Lebanon, NH, USA) in 200 µL of PBS at day 10 and 15 after AOM/DSS administration. CAC induction and progression were analyzed as mentioned in Section 4.2.

### 4.7. Statistical Analysis

Data were analyzed by one-way analysis of variance followed by Tukey’s multiple comparisons test or unpaired two-tailed t-tests depending on the number of groups using GraphPad Prism 5 (San Diego, CA, USA). All statistical tests were performed considering 95% confidence intervals. The data are expressed as the mean ± S.E. * = *p* < 0.05, ** = *p* < 0.01.

## Data Availability

Not applicable.

## References

[1] Siegel R., DeSantis C., Jemal A. (2014). Colorectal cancer statistics, 2014. CA Cancer J. Clin..

[2] Sung H., Ferlay J., Siegel R.L., Laversanne M., Soerjomataram I., Jemal A., Bray F. (2021). Global cancer statistics 2020: GLOBOCAN estimates of incidence and mortality worldwide for 36 cancers in 185 countries. CA Cancer J. Clin..

[3] Colotta F., Allavena P., Sica A., Garlanda C., Mantovani A. (2009). Cancer-related inflammation, the seventh hallmark of cancer: Links to genetic instability. Carcinogenesis.

[4] Van Der Kraak L., Gros P., Beauchemin N. (2015). Colitis-associated colon cancer: Is it in your genes?. World J. Gastroenterol..

[5] Hebenstreit D., Wirnsberger G., Horejs-Hoeck J., Duschl A. (2006). Signaling mechanisms, interaction partners, and target genes of STAT6. Cytokine Growth Factor Rev..

[6] Delgado-Ramirez Y., Colly V., Gonzalez G.V., Leon-Cabrera S. (2020). Signal transducer and activator of transcription 6 as a target in colon cancer therapy (Review). Oncol. Lett..

[7] Andre F., Arnedos M., Baras A.S., Baselga J., Bedard P.L., Berger M.F., Bierkens M., Calvo F., Cerami E., Chakravarty D. (2017). AACR Project GENIE: Powering Precision Medicine through an International Consortium. Cancer Discov..

[8] Uhlén M., Zhang C., Lee S., Sjöstedt E., Fagerberg L., Bidkhori G., Benfeitas R., Arif M., Liu Z., Edfors F. (2017). A pathology atlas of the human cancer transcriptome. Science.

[9] Wang C.-G., Ye Y.-J., Yuan J., Liu F.-F., Zhang H., Wang S. (2010). EZH2 and STAT6 expression profiles are correlated with colorectal cancer stage and prognosis. World J. Gastroenterol..

[10] Wick E.C., Leblanc R.E., Ortega G., Robinson C., Platz E., Pardoll E.M., Iacobuzio-Donahue C., Sears C.L. (2012). Shift from pStat6 to pStat3 predominance is associated with inflammatory bowel disease-associated dysplasia. Inflamm. Bowel Dis..

[11] Li B.H., Yang X.Z., Li P.D., Yuan Q., Liu X.H., Yuan J., Zhang W.J. (2008). IL-4/Stat6 activities correlate with apoptosis and metastasis in colon cancer cells. Biochem. Biophys. Res. Commun..

[12] Lin Y., Li B., Yang X., Liu T., Shi T., Deng B., Zhang Y., Jia L., Jiang Z., He R. (2019). Non-hematopoietic STAT6 induces epithelial tight junction dysfunction and promotes intestinal inflammation and tumorigenesis. Mucosal Immunol..

[13] Leon-Cabrera S.A., Molina-Guzman E., Delgado-Ramirez Y.G., Vázquez-Sandoval A., Ledesma-Soto Y., Pérez-Plasencia C.G., Chirino Y.I., Delgado-Buenrostro N.L., Rodríguez-Sosa M., Vaca-Paniagua F. (2017). Lack of STAT6 Attenuates Inflammation and Drives Protection against Early Steps of Colitis-Associated Colon Cancer. Cancer Immunol. Res..

[14] Olguín J.E., Medina-Andrade I., Rodríguez T., Rodríguez-Sosa M., Terrazas L.I. (2020). Relevance of Regulatory T Cells during Colorectal Cancer Development. Cancers.

[15] Liu Z., Huang Q., Liu G., Dang L., Chu D., Tao K., Wang W. (2014). Presence of FOXP3+Treg cells is correlated with colorectal cancer progression. Int. J. Clin. Exp. Med..

[16] Argon A., Vardar E., Kebat T., Ömer E., Erkan N. (2016). The Prognostic Significance of FoxP3+ T Cells and CD8+ T Cells in Colorectal Carcinomas. J. Environ. Pathol. Toxicol. Oncol..

[17] Reimers M.S., Engels C.C., Putter H., Morreau H., Liefers G.J., Van De Velde C.J.H., Kuppen P.J.K. (2014). Prognostic value of HLA class I, HLA-E, HLA-G and Tregs in rectal cancer: A retrospective cohort study. BMC Cancer.

[18] Soh J.S., Jo S.I., Lee H., Do E.-J., Hwang S.W., Park S.H., Ye B.D., Byeon J.-S., Yang S.-K., Kim J.H. (2019). Immunoprofiling of Colitis-associated and Sporadic Colorectal Cancer and its Clinical Significance. Sci. Rep..

[19] Vlad C., Kubelac P., Fetica B., Vlad D., Irimie A., Achimas-Cadariu P. (2015). The prognostic value of FOXP3+ T regulatory cells in colorectal cancer. J. BUON.

[20] Saito T., Nishikawa H., Wada H., Nagano Y., Sugiyama D., Atarashi K., Maeda Y., Hamaguchi M., Ohkura N., Sato E. (2016). Two FOXP3+CD4+ T cell subpopulations distinctly control the prognosis of colorectal cancers. Nat. Med..

[21] Olguín J.E., Medina-Andrade I., Molina E., Vázquez A., Pacheco-Fernández T., Saavedra R., Pérez-Plasencia C., Chirino Y.I., Vaca-Paniagua F., Arias-Romero L.E. (2018). Early and Partial Reduction in CD4+Foxp3+ Regulatory T Cells during Colitis-Associated Colon Cancer Induces CD4+ and CD8+ T Cell Activation Inhibiting Tumorigenesis. J. Cancer.

[22] Bruns H.A., Schindler U., Kaplan M.H. (2003). Expression of a Constitutively Active Stat6 In Vivo Alters Lymphocyte Homeostasis with Distinct Effects in T and B Cells. J. Immunol..

[23] Sanchez-Guajardo V., Tanchot C., O’Malley J.T., Kaplan M.H., Garcia S., Freitas A.A. (2007). Agonist-driven development of CD4+CD25+Foxp3+ regulatory T cells requires a second signal mediated by Stat6. J. Immunol..

[24] Zorn E., Nelson E.A., Mohseni M., Porcheray F., Kim H., Litsa D., Bellucci R., Raderschall E., Canning C., Soiffer R.J. (2006). IL-2 regulates FOXP3 expression in human CD4+CD25+ regulatory T cells through a STAT-dependent mechanism and induces the expansion of these cells in vivo. Blood.

[25] Takaki H., Ichiyama K., Koga K., Chinen T., Takaesu G., Sugiyama Y., Kato S., Yoshimura A., Kobayashi T. (2008). STAT6 inhibits TGF-beta 1-mediated Foxp3 induction through direct binding to the Foxp3 promoter, which is reverted by retinoic acid receptor. J. Biol. Chem..

[26] Dorsey N.J., Chapoval S.P., Smith E.P., Skupsky J., Scott D.W., Keegan A.D. (2013). STAT6 controls the number of regulatory T cells in vivo, thereby regulating allergic lung inflammation. J. Immunol..

[27] Setiady Y.Y., Coccia J.A., Park P.U. (2010). In vivo depletion of CD4(+)FOXP3(+) Treg cells by the PC61 anti-CD25 monoclonal antibody is mediated by Fc gamma RIII+ phagocytes. Eur. J. Immunol..

[28] Rosen M.J., Chaturvedi R., Washington M.K., Kuhnhein L.A., Moore P.D., Coggeshall S.S., McDonough E.M., Weitkamp J.-H., Singh A.B., Coburn L.A. (2013). STAT6 deficiency ameliorates severity of oxazolone colitis by decreasing expression of claudin-2 and Th2-inducing cytokines. J. Immunol..

[29] Jayakumar A., Bothwell A.L. (2017). Stat6 Promotes Intestinal Tumorigenesis in a Mouse Model of Adenomatous Polyposis by Expansion of MDSCs and Inhibition of Cytotoxic CD8 Response. Neoplasia.

[30] Vignali D.A.A., Collison L.W., Workman C.J. (2008). How regulatory T cells work. Nat. Rev. Immunol..

[31] Ling Z.-A., Zhang L.-J., Ye Z.-H., Dang Y.-W., Chen G., Li R.-L., Zeng J.-J. (2018). Immunohistochemical distribution of FOXP3+ regulatory T cells in colorectal cancer patients. Int. J. Clin. Exp. Patho..

[32] Ma Q., Liu J., Wu G., Teng M., Wang S., Cui M., Li Y. (2018). Co-expression of LAG3 and TIM3 identifies a potent Treg population that suppresses macrophage functions in colorectal cancer patients. Clin. Exp. Pharmacol. Physiol..

[33] Norton S.E., Ward-Hartstonge K.A., McCall J.L., Leman J.K.H., Taylor E.S., Munro F., Black M.A., Groth B.F.D.S., McGuire H.M., Kemp R.A. (2019). High-Dimensional Mass Cytometric Analysis Reveals an Increase in Effector Regulatory T Cells as a Distinguishing Feature of Colorectal Tumors. J. Immunol..

[34] Clarke S.L., Betts G.J., Plant A., Wright K.L., El-Shanawany T.M., Harrop R., Torkington J., Rees B.I., Williams G.T., Gallimore A.M. (2006). CD4+CD25+FOXP3+ Regulatory T Cells Suppress Anti-Tumor Immune Responses in Patients with Colorectal Cancer. PLoS ONE.

[35] Hanke T., Melling N., Simon R., Sauter G., Bokemeyer C., Lebok P., Terracciano L.M., Izbicki J.R., Marx A.H. (2015). High intratumoral FOXP3+ T regulatory cell (Tregs) density is an independent good prognosticator in nodal negative colorectal cancer. Int. J. Clin. Exp. Pathol..

[36] Shang B., Liu Y., Jiang S.J., Liu Y. (2015). Prognostic value of tumor-infiltrating FoxP3(+) regulatory T cells in cancers: A systematic review and meta-analysis. Sci. Rep..

[37] Saito T., Yamashita K., Tanaka K., Yamamoto K., Makino T., Takahashi T., Kurokawa Y., Yamasaki M., Wada H., Nishikawa H. (2021). Impact of tumor infiltrating effector regulatory T cells on the prognosis of colorectal cancers. Cancer Sci..

[38] Fantini M.C., Favale A., Onali S., Facciotti F. (2020). Tumor Infiltrating Regulatory T Cells in Sporadic and Colitis-Associated Colorectal Cancer: The Red Little Riding Hood and the Wolf. Int. J. Mol. Sci..

[39] Omenetti S., Pizarro T.T. (2015). The Treg/Th17 Axis: A Dynamic Balance Regulated by the Gut Microbiome. Front. Immunol..

[40] Liu Y.-J., Tang B., Wang F.-C., Tang L., Lei Y.-Y., Luo Y., Huang S.-J., Yang M., Wu L.-Y., Wang W. (2020). Parthenolide ameliorates colon inflammation through regulating Treg/Th17 balance in a gut microbiota-dependent manner. Theranostics.

[41] Ladoire S., Martin F., Ghiringhelli F. (2011). Prognostic role of FOXP3+ regulatory T cells infiltrating human carcinomas: The paradox of colorectal cancer. Cancer Immunol. Immunother..

[42] Maloy K.J., Salaun L., Cahill R., Dougan G., Saunders N.J., Powrie F. (2003). CD4(+)CD25(+) T-R cells suppress innate immune pathology through cytokine-dependent mechanisms. J. Exp. Med..

[43] Mottet C., Uhlig H.H., Powrie F. (2003). Cutting Edge: Cure of Colitis by CD4+CD25+ Regulatory T Cells. J. Immunol..

[44] Watanabe K., Rao V.P., Poutahidis T., Rickman B.H., Ohtani M., Xu S., Rogers A.B., Ge Z., Horwitz B.H., Fujioka T. (2008). Cytotoxic-T-Lymphocyte-Associated Antigen 4 Blockade Abrogates Protection by Regulatory T Cells in a Mouse Model of Microbially Induced Innate Immune-Driven Colitis. Infect. Immun..

[45] Canavan J.B., Scotta C., Vossenkamper A., Goldberg R., Elder M.J., Shoval I., Marks E., Stolarczyk E., Lo J.W., Powell N. (2016). Developing in vitro expanded CD45RA(+) regulatory T cells as an adoptive cell therapy for Crohn’s disease. Gut.

[46] Clough J.N., Omer O.S., Tasker S., Lord G.M., Irving P.M. (2020). Regulatory T-cell therapy in Crohn’s disease: Challenges and advances. Gut.

[47] Desalegn G., Pabst O. (2019). Inflammation triggers immediate rather than progressive changes in monocyte differentiation in the small intestine. Nat. Commun..

[48] Chu K.-H., Lin S.-Y., Chiang B.-L. (2021). STAT6 Pathway Is Critical for the Induction and Function of Regulatory T Cells Induced by Mucosal B Cells. Front. Immunol..

[49] Leon-Cabrera S., Vázquez-Sandoval A., Molina-Guzman E., Delgado-Ramirez Y., Delgado-Buenrostro N.L., Callejas B.E., Chirino Y.I., Pérez-Plasencia C., Rodríguez-Sosa M., Olguín J.E. (2018). Deficiency in STAT1 Signaling Predisposes Gut Inflammation and Prompts Colorectal Cancer Development. Cancers.

